# A community-wide acute diarrheal disease outbreak associated with drinking contaminated water from shallow bore-wells in a tribal village, India, 2017

**DOI:** 10.1186/s12889-020-8263-2

**Published:** 2020-02-14

**Authors:** Kiran Kumar Maramraj, G. Subbalakshmi, Mohammed Shahed Ali, Tanzin Dikid, Rajesh Yadav, Samir V. Sodha, Sudhir Kumar Jain, Sujeet Kumar Singh

**Affiliations:** 1 National Centre for Disease Control, 22 Sham Nath Marg, New Delhi, 110054 India; 2 State health department, Hyderabad, Telangana 500095 India; 3 Division of Global Health Protection, United States Centers for Disease Control and Prevention, New Delhi, India; 4 Division of Global Health Protection, United States Centers for Disease Control and Prevention, Atlanta, USA

**Keywords:** Acute diarrheal disease, Outbreak, Bore-well, Tribal

## Abstract

**Background:**

In 2016, India reported 709 acute diarrheal disease (ADD) outbreaks (> 25% of all outbreaks). Tribal populations are at higher risk with 27% not having accessibility to safe drinking water and 75% households not having toilets. On June 26, 2017 Pedda-Gujjul-Thanda, a tribal village reported an acute diarrheal disease (ADD) outbreak. We investigated to describe the epidemiology, identify risk factors, and provide evidence-based recommendations.

**Methods:**

We defined a case as ≥3 loose stools within 24 h in Pedda-Gujjul-Thanda residents from June 24–30, 2017. We identified cases by reviewing hospital records and house-to-house survey. We conducted a retrospective cohort study and collected stool samples for culture. We assessed drinking water supply and sanitation practices and tested water samples for faecal-contamination.

**Results:**

We identified 191 cases (65% females) with median age 36 years (range 4–80 years) and no deaths. The attack-rate (AR) was 37% (191/512). Downhill colonies (located on slope of hilly terrains of the village) reported higher ARs (56%[136/243], *p* < 0.001) than others (20%[55/269]). Symptoms included diarrhea (100%), fever (17%), vomiting (16%) and abdominal pain (13%). Drinking water from five shallow bore-wells located in downhill colonies was significantly associated with illness (RR = 4.6, 95%CI = 3.4–6.1 and population attributable fraction 61%). In multi-variate analysis, drinking water from the shallow bore-wells located in downhill colonies (aOR = 7.9, [95% CI =4.7–13.2]), illiteracy (aOR =6, [95% CI = 3.6–10.1]), good hand-washing practice (aOR = 0.4, [95%CI = 0.2–0.7]) and household water treatment (aOR = 0.3, [95%CI = 0.2–0.5]) were significantly associated with illness. Two stool cultures were negative for *Vibrio cholerae*. Heavy rainfall was reported from June 22–24. Five of six water samples collected from shallow bore-wells located in downhill colonies were positive for faecal contamination.

**Conclusion:**

An ADD outbreak with high attack rate in a remote tribal village was associated with drinking water from shallow downhill bore-wells, likely contaminated via runoff from open defecation areas after heavy rains. Based on our recommendations, immediate public health actions including repair of leakages at contaminated water sources and alternative supply of purified canned drinking water to families, and as long-term public health measures construction of house-hold latrines and piped-water supply initiated.

## Introduction

Globally there are an estimated 1.7 billion cases and 2.2 million deaths from acute diarrheal disease (ADD) every year [1]. In India, the burden is particularly high with more than 13.9 million cases reported in 2016 and 709 ADD outbreaks reported accounting to more than 25% of all outbreaks [2, 3].

Lack of access to safe drinking water and basic sanitation are the leading causes of ADD burden globally and in India. It is estimated that globally 58% of ADD deaths are attributed to inadequate drinking water, sanitation and hygiene [4]. The WHO/UNICEF Joint Monitoring Program for Water Supply, Sanitation and Hygiene (JMP) 2017 report revealed that 844 million people worldwide lack access to basic drinking-water service and 2.3 billion lack basic sanitation services, while 892 million still practiced open defecation [5]. The National Family Health Survey (NFHS-4, 2015–16) reported that in India only 52% of urban households and 18% of rural households have piped water supply, and the main source of water supply among rural households is bore-wells or tube-wells (51%). It has been estimated that 39% of households in India (54% among rural households) have no toilet facility and practicing open defecation [6].

The “indigenous” populations are socially, culturally and economically isolated and usually lack access to basic drinking-water and sanitation services. Therefore, they are vulnerable to ADD outbreaks and other emerging and re-emerging diseases [7]. The United Nations estimates that there are 370 million indigenous people existing across 90 countries of the world. They constitute 5% of the world population but 15% of the poorest [8]. India alone houses more than 705 such indigenous groups termed as Scheduled Tribes. As per the Census 2011, the total Scheduled Tribe population of India is 10.43 crore with a significant proportion of them living in rural areas [9].

On June 26, 2017, Kama-reddy district of Telangana state reported 55 ADD cases from the Pedda-Gujjul-Thanda village. We conducted the outbreak investigation to describe the epidemiology, identify risk factors, and provide evidence-based recommendations.

## Methods

### Setting

Pedda-Gujjul-Thanda village is a small tribal village with a total population of 563. The village is remotely located as an isolated community with a hilly terrain and is resource-limited with poor accessibility to sanitation and hygiene facilities. The nearest health care facility available for the residents is located at a distance of 10 km from the village.

### Case definition

We defined a case as three or more loose stools within 24 h in a resident of the Pedda-Gujjul-Thanda village from June 22, 2017 to July 2, 2018.

### Case finding

To find cases, we reviewed medical records of local health care facilities accessed by village residents in the nearby town. We conducted a medical camp in the village during the outbreak period for five days. We conducted a house-to-house survey in the village to find more cases, which are niether reported to health facility nor medical camp.

### Retrospective cohort study

We conducted a retrospective cohort study to identify risk factors associated with illness. We defined the cohort as residents of Pedda-Gujjul-Thanda village from June 22, 2017 to July 2, 2018. Village resident was the unit of analysis. For data collection, we trained five teams of local paramedical staff. Using a pre-structured questionnaire, we collected data on demographic characteristics and risk factors related to drinking water, sanitation and hygiene. Good hand-washing practice was defined as reported washing of hands with soap and water every time after defecation and before eating. A bore-well less than 30-m-deep, as assessed from the records of village administration, was considered a shallow bore-well.

### Laboratory and environmental investigations

Two stool samples were collected by the treating physician from admitted patients on the first day of hospital admission and transported to the state reference laboratory within two hours in Cary-Blair transport medium. The samples were cultured for *Vibrio cholerae, Salmonella* and *Shigella* on nutrient agar, MacConkey agar and deoxycholate citrate agar. Enteric pathogens were identified by biochemical reaction and by agglutination with anti-sera. We collected details of recent rainfall and conducted an environmental survey with household as sampling unit to assess drinking water, sanitation and hygiene practices. We assessed availability of residual chlorine in all village bore-wells and tested four of five bore-wells in the most affected colonies for faecal contamination by H_2_S method in field. Water was filled up to the ‘fill line’ of the sample bottle and incubated at room temperature (25^0^–37^0^ C) for 36–48 h and observed for colour change in the medium. A water sample was suspected to be contaminated with faecal matter, if it turned black [10, 11]. Because of limited supplies, we were unable to assess the fifth bore-well.

### Data analysis

We analysed the data to describe the occurrence of cases over time, place, and person. We calculated relative risks (RR) with 95% confidence intervals (CI), population attributable risk percentages and conducted multiple logistic regression analysis with the dependent variables including consumption of shallow-downhill bore-well water, report of visible contaminants like mud in drinking water, illiteracy, household water treatment and good hand-washing practice. We used Epi Info version 7.2 for statistical analysis.

## Results

### Descriptive epidemiology

We identified 191 ADD cases (65% females), with a village attack rate (AR) of 37% (191/512). The attack rate increased with age, with highest among > 60-year age group (55%) and lowest among children under-10 years (11%) (Table 1). No deaths were reported.

**Table 1:** Attack-rates in an Acute Diarrheal Disease outbreak by age and sex in Pedda-Gujjula-Thanda village, 2017

	Number of cases	Population	Attack rate (%)
Age Group (years)			
Children (0–9)	12	105	11
Young adults (10–19)	23	77	29
Adults (20–59)	125	274	46
20–29	39	97	40
30–39	42	84	50
40–49	29	60	48
50–59	15	33	45
Elderly (≥60)	31	56	55
Gender			
Male	67	246	27
Female	124	266	47
Overall Incidence	191	512^a^	37

^a^Out of 563 population, 512 were present during outbreak period

In addition to diarrhea, cases presented with fever (17%), vomiting (16%) and abdominal pain (13%). 72% (138/191) cases reported to health care facilities and the medical camp conducted in the village. Among the 191 cases, 159 (83%) had mild illness treated with oral rehydration solution; 30 (16%) had moderate dehydration treated with intravenous fluids on out-patient basis, and 2 (1%) with severe dehydration were admitted in the district hospital for treatment with antibiotics (metronidazole and ciprofloxacin) and intravenous fluids.

**Fig. 1:**
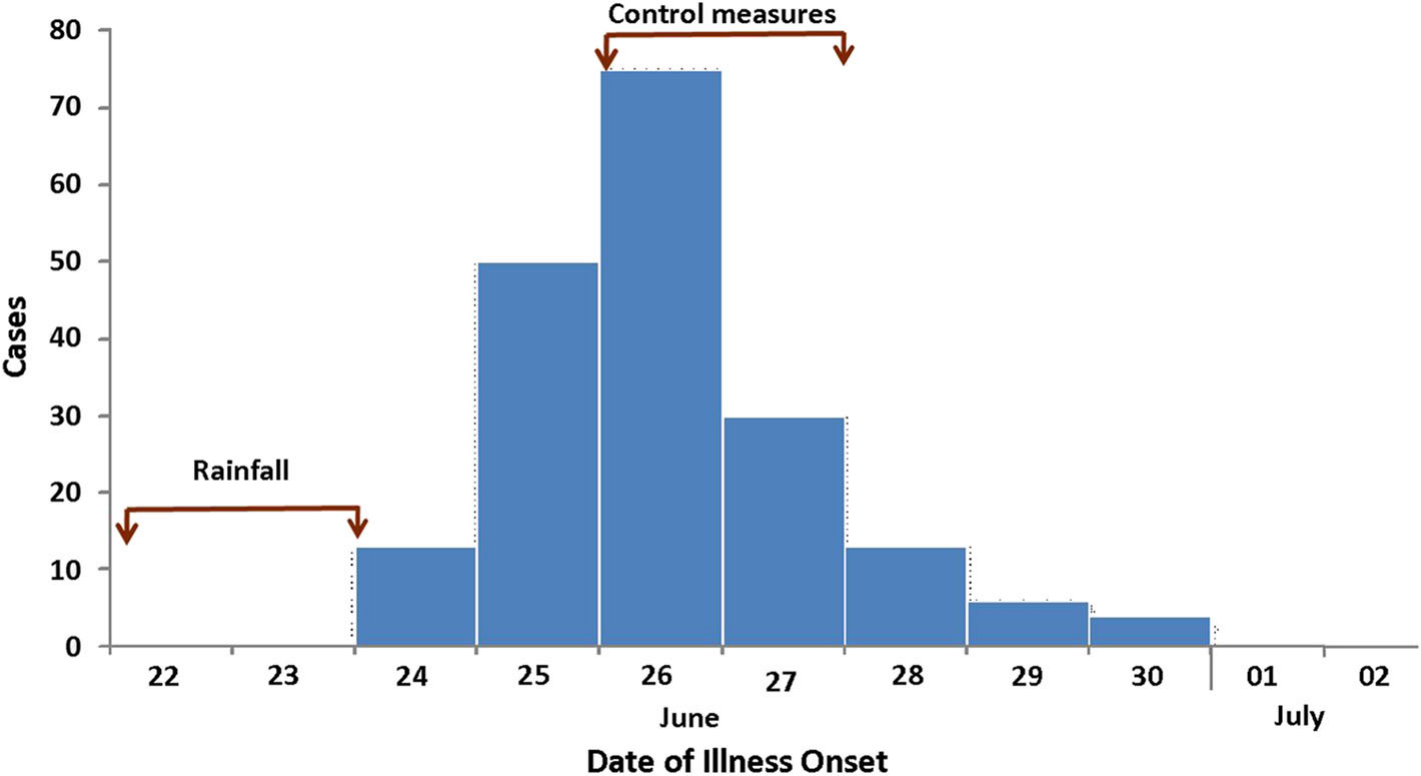
Distribution of cases by date of illness onset in an Acute Diarrheal Disease outbreak in Pedda-Gujjula-Thanda Village, 2017 (*n* = 191)*

Cases started reported on June 26, 2017, with onset of symptoms from 24 June 2017. Maximum cases were reported on June 27, 2017, and no new cases were reported after June 30, 2017 (Fig. 1).

**Fig. 2:**
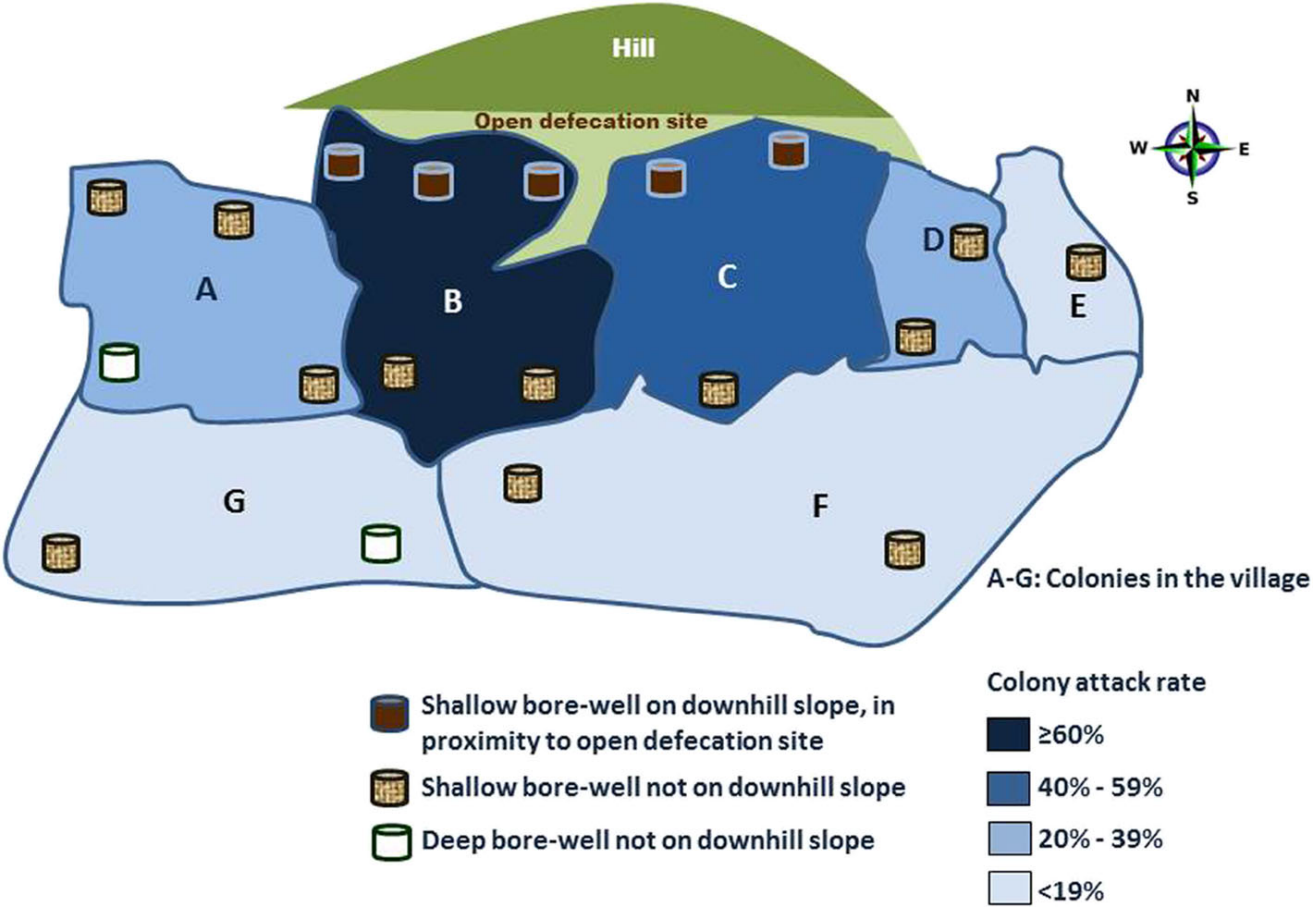
Area map showing bore-wells, open-defecation site and colony-wise attack-rates in Acute Diarrheal Disease outbreak in Pedda-Gujjula-Thanda Village, 2017*

The tribal population in the village had four sub-tribes namely *Katroth, Badhawath, Nenawath, Baromath* who resided in seven geographically demarcated colonies (labelled as A to G). *Katroth* sub-tribe resided in colonies A, B and G; *Badhawath* in colonies C and D; *Nenawath* in colony E and *Baromath* in colony F (Table 2). Colonies B and C had higher attack rates (65 and 47% respectively) as compared to other colonies (Fig. 2).

**Table 2:** Distribution of Acute Diarrheal Disease outbreak by colony in Pedda-Gujjula-Thanda tribal village, 2017

Colony	Distribution of cases	Attack rates
B (*Katroth*-3)^a^	79 (41.4%)	65%
C (*Badhawath*-1)^a^	57 (29.8%)	47%
A (*Katroth*-2)	26 (13.6%)	35%
D (*Badhawath*-2)	9 (4.7%)	23%
F (*Baromath*)	9 (4.7%)	17%
E (*Nenawath*)	6 (3.1%)	13%
G (*Katroth*-1)	5 (2.6%)	9%
Total	191 (100%)	37%

^a^B and C colonies are downhill colonies located on slope of hilly terrain

### Retrospective cohort study

Among 563 village residents, 512 (91%) participated in the study. Among the 512 participants, median age was 28 years (range 1–80 years) with 52% females; 50% reported as illiterate with agriculture as the main source of livelihood for 76%.

We analysed possible risk factors associated with ADD (Table 3). Drinking water from bore-well groundwater (vs canned water) was found significantly associated with ADD (RR = 12.7; 95% CI = 1.8–87.4). However, only 32 (6%) residents in the village used canned water and bore-well groundwater was the predominant source of water supply. Therefore, we analysed the water sources further, by location and type of bore-wells. Residents who used any of the five shallow bore-wells located downhill were significantly at higher risk (RR = 4.6; 95% CI = 3.4–6.1) and deep bore-wells were protective (RR = 0.4; 95% CI = 0.2–0.9). Report of visible contaminants like mud in drinking water (aOR = 4; 95% CI = 2.1–7.6) and illiteracy (aOR = 3.6; 95% CI = 3.5–10.1) were significantly associated with illness; and household water treatment (done either by boiling or use of candle filters) (aOR = 0.4; 95% CI = 0.2–0.7) and good hand-washing practice (aOR = 0.2; 95% CI = 0.1–0.5) were found protective.

**Table 3:** Risk factors associated with Acute Diarrheal Disease outbreak in Pedda-Gujjula-Thanda tribal village, 2017 (*n* = 512)

Risk Factor	Attack rate Exposed	Attack rate Non-exposed	RR (95% CI)	MLR analysis* Adjusted OR (95% CI)
Consumption of bore-well water source (vs canned water)	190/480 (40%)	1/32 (3%)	12.7 (1.8–87.4)	Excluded^‡^
Consumption of shallow-downhill bore-well water (vs all other drinking water sources) ^†^	149/224 (67%)	42/288 (15%)	4.6 (3.4–6.1)	7.9 (4.7–13.2)
Report of visible contaminants like mud in drinking water	65/94 (69%)	126/418 (30%)	2.3 (1.9–2.8)	4.0 (2.1–7.6)
Practice of open defecation	186/484 (38%)	5/28 (18%)	2.2 (1.0–4.8)	Excluded^§^
Illiteracy^¶^	131/252 (52%)	52/188 (28%)	1.9 (1.4–2.4)	3.6 (3.6–10.1)
Kutcha house type	174/457 (38%)	17/55 (31%)	1.2 (0.8–1.9)	Excluded^§^
Housefly menace (Flies sighted in the house during the minimum 15 min-visit)	39/95 (41%)	152/417 (36)	1.1 (0.9–1.5)	Excluded^§^
Recent accumulation of water in or around house	73/182 (40%)	118/330 (36)	1.1 (0.8–1.4)	Excluded^§^
Use of narrow mouth container for household water storage	29/80 (36%)	162/432 (38%)	0.9 (0.7–1.3)	Excluded^§^
Deep bore-wells (vs all other drinking water sources) ^†^	6/38 (16%)	185/474 (39%)	0.4 (0.2–0.9)	Excluded^‖^
Household water treatment	24/154 (16%)	167/358 (47%)	0.3 (0.2–0.5)	0.4 (0.2–0.7)
Good hand-washing practice	11/101 (11%)	180/411 (44%)	0.2 (0.1–0.4)	0.2 (0.1–0.5)

*Multiple logistic regression analysis

^**†**^Five of 19 bore-wells were shallow and located on downhill slope, in close proximity to open defecation site; and two of 19 bore-wells were deep type

^**‡**^Excluded from MLR analysis due to relatively negligible sample size of residents using canned water with a very broad 95% CI in bivariate analysis

^**§**^Excluded from MLR analysis as the variable did not meet the statistical inclusion criteria (*p* < 0.25)

^‖^Excluded from MLR analysis as the variable is multi-collinear with other bore-well variables

^¶^Less than seven-year-old excluded (*n* = 440)

### Laboratory and environmental results

**Fig. 3:**
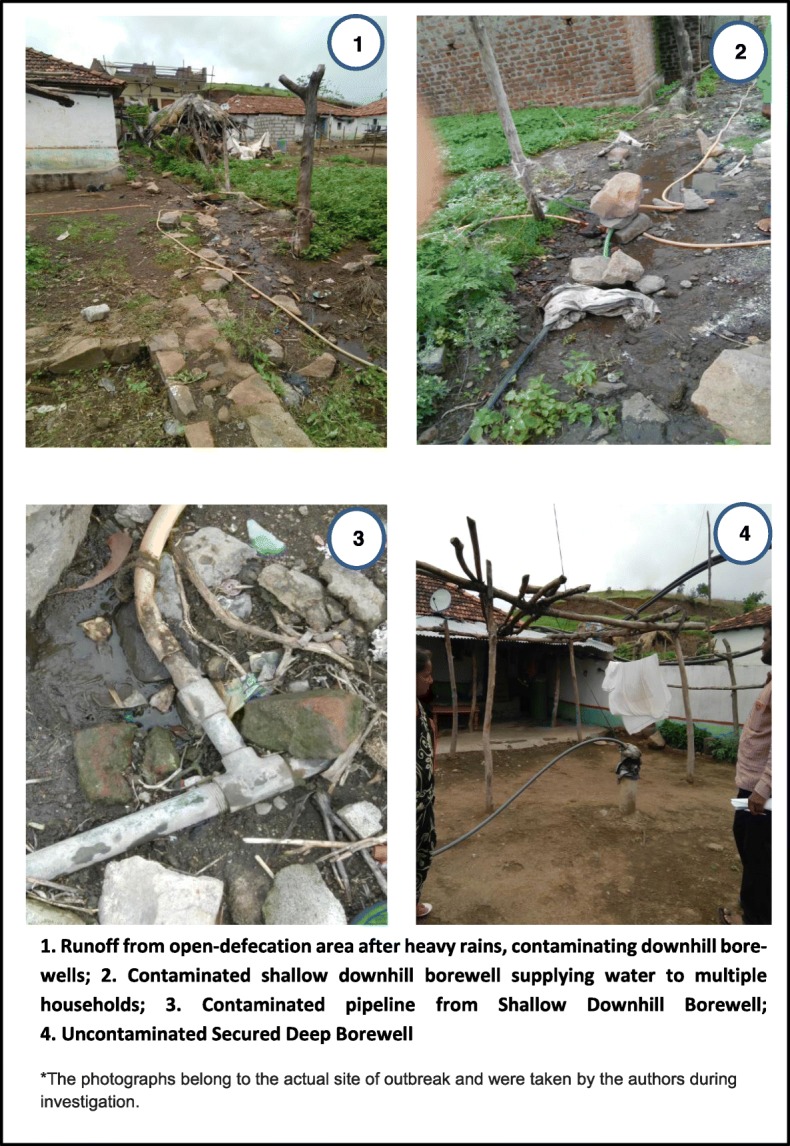
Photographs of Open-Defecation site, Downhill Shallow Bore-wells and a Deep Borewell, in Acute Diarrheal Disease Outbreak in Pedda-Gujjula-Thanda Village, 2017*

Stool samples collected from two hospitalized cases showed no growth for *Vibrio cholerae*, *Salmonella* and *Shigella* on culture. Among 110 households, 100 (91%) were available for environmental survey. Among the 100 houses surveyed, 79 (79%) were *kutcha* (low quality) type, made of mud, thatch and other low-quality material. Only 5 (5%) households had a designated toilet at home while the remaining 95 (95%) practiced open defecation at a site located on the slope of the hill behind the downhill colonies B and C (Figs. 2 and 3). Bore-wells were the main source of drinking water supply for 93 (93%) households. There were two deep bore-wells provided by the village administration and 17 shallow type bore-wells privately constructed by village residents. Five of these 17 (30%) shallow bore-wells were located in colonies B and C, on the downhill slope below the open defecation site. Plastic pipelines from the shallow wells were improperly installed with leakages at multiple points. There was no facility at source, for chlorination or any other mode of purification. Thirty households (30%) treated the water before consumption either by boiling or by use of candle filters. There was no routine drinking water surveillance in place by any authority for assessing the quality and fitness for drinking water. There was no residual chlorine found in any water samples. Three of four drinking water samples from bore-wells of most affected colonies (B and C) indicated faecal contamination by H_2_S field testing. There was heavy rainfall (average 65 mm in a day) from 22 to 24 June 2017.

### Prevention and control measures undertaken to contain the outbreak

The village residents were discouraged from using shallow bore-well water and were provided with safe canned drinking water until all leakages were secured. Leakages in water supply from the bore-wells were identified and secured. Chlorine tablets were distributed for household level water disinfection. We informed the residents to avoid open defecation near drinking water sources and residential premises. Public health staff conducted health education daily to improve awareness among the villagers about water, sanitation, and hygiene. After active implementation of these control measures, cases declined rapidly in the village (Fig. 1).

## Discussion

A rapid systematic epidemiological investigation of this outbreak identified water contamination points and likely mode of contamination. Based on these findings and our recommendations, the local health department instituted immediate public health actions including repair of leakages at contaminated water sources and alternative supply of purified canned drinking water to families. Effective implementation of public health measures limited the exposure of the community to contaminated water source resulting in rapid containment of the outbreak.

Waterborne disease outbreaks tend to have cases spread over a time-period due to ongoing exposure to the contaminated water. In contrast, the pattern of epidemic curve in the present waterborne disease outbreak resembled that of food-borne with a point source exposure. Heavy rains contributed to the run-off of water from the open defecation site into the ground water of shallow wells located on slopes of hilly terrain resulting in heavy contamination and sudden rise of cases. Rapid control measures in the small village, implemented effectively within a short period of time, may have led to rapid decline of cases. The available epidemiological evidence also did not support generation of hypothesis of food-borne origin of the outbreak. In an outbreak reported among school children in Northern Greece in 2012, investigation revealed a waterborne viral gastroenteritis outbreak with a point source pattern, due to consumption of heavily contaminated water from a tap, which was not in use for two weeks during Christmas vacation [12].

Attack rate was high in this outbreak (37%), possibly due to exposure to high pathogen load subsequent to gross faecal contamination of water sources. In the absence of other alternative water sources, this tribal community was exclusively dependent on the contaminated water source for drinking, therefore exposing a large section of the community to risk. Geetha et al. analysed 32 diarrheal outbreaks in south India in non- tribal communities and reported lower attack rates varying from 0.6 to 21.5% [13]. However, tribal populations in India such as in Pedda-Gujjul-Thanda are marginalized with poor availability of WASH facilities [14]. This vulnerable tribal population continues to be at higher risk for ADD outbreaks with 27% not having access to safe drinking water and 75% of households not having toilets [15]. They need special assistance schemes from the government to enable them overcome poor accessibility to WASH facilities and secure healthy living [16].

Due to inadequate availability of communally managed safe public water points by the local authority, this community in Pedda-Gujjul-Thanda village was dependent on privately constructed shallow bore-wells for water supply. These are economical but likely to be unsafe. In this outbreak, open defecation site was present on the downhill slope in proximity to the residential premises and water resources, increasing the risk of drinking water contamination. Among the entire village population, 61% of ADD cases were attributable to drinking water from the ‘shallow downhill bore-wells’ (Population Attributable Fraction 61%), which was also evident from rapid outbreak containment following the elimination of exposure to this single risk factor. Since this exposure factor is amenable to long-term public health intervention, permanent elimination of shallow downhill bore-wells as water source was recommended, replacing them with properly secured deep bore-wells.

Shallow bore-wells are known for their susceptibility to contamination from surface land-use activities [17, 18]. Studies have found levels of *E. coli* and enteric viruses to be high in shallow sources of ground water especially when they are in close proximity to polluting sources [19–21]. Consumption of ground water from shallow bore-wells with no purification facility increases the risk of diarrhea outbreaks manifold [22, 23]. A meta-analytic study of water-borne diarrheal disease outbreaks in China reported that 78 of 85 (92%) outbreaks (between year 1987 to 2014) were due to poor sanitary conditions of wells with lavatories/septic tanks nearby and lack of purification facilities [24]. In developed countries and urban areas of developing countries, as water supply and sanitation have improved dramatically over a period of time, such outbreaks were rarely reported in the recent past. The largest *E. coli* O157 outbreak in United States occurred in 1999 at a county fair (781 ill persons and 2 deaths) was due to groundwater source from a temporary unregulated well at the fairground [25].

Our findings have implication for India’s progress towards United Nation’s Sustainable Development Goal (SDG) 6 and India’s nation-wide campaign ‘*Swachh Bharat Mission* (SBM)’ to ensure availability and management of water and sanitation for all. SDG 6 aims at achieving universal access to basic sanitation service by 2030; and it has been reported that between 2000 and 2017, the proportion lacking even a basic sanitation service decreased from 44 to 27% [26]. SBM aims to achieve an “open-defecation free” status in rural areas through the construction of household-owned and community-owned toilets and establishing an accountable mechanism of monitoring toilet use. In 2015 in India, around 524 million (39%) practiced open defecation. However, under the SBM mission, due to increase in ‘households with toilets’ only 19 million (1.4%) practiced open defecation in January 2019 [5, 27]. There has also been a 71.58% increase in ‘households with toilets’ from October 2014 to October 2019 in rural areas of the Telangana state in India [27].

The tribal community initially obstructed the effective delivery of health care services; however, after involvement of the local stakeholders and tribal leaders, the acceptance towards medical treatment and community health services improved. Notwithstanding, most of the patients were still reluctant and did not consent for giving stool specimens for laboratory diagnosis. Establishing a rapport with the reticent tribal community was a major challenge faced by the outbreak investigation team. Lack of microbiological aetiology confirmation of the outbreak remained a limitation of the investigation due to limited stool samples and laboratory-capacity constraints of the remote area.

Recognizing the pivotal importance of SDGs, national health policy of India (2017) has set the health-related cross-sectorial goal “access to safe water and sanitation to all by 2020” [28]. Greater political and financial commitment towards resource-limited remote tribal areas with effective community mobilization is required to accelerate the public health interventions to improve WASH and to prevent ADD outbreaks in the future.

## Conclusion

This was a community-wide acute diarrheal disease outbreak with high village attack rate in a remote tribal village of Telangana with poor availability of safe water, sanitation and hygiene (WASH) facilities. A rapid and systematic epidemiological investigation identified drinking of faecal-contaminated water from the shallow bore-wells as the leading cause for this outbreak. These bore-wells were likely contaminated from runoff after rain from open defecation areas located on a downhill slope. Prompt and targeted public health action contained the number of cases.

## Data Availability

The datasets used and/or analysed during the current study are available from the corresponding author on reasonable request.

## Abbreviations

ADD: Acute Diarrheal Disease
H2S: Hydrogen Sulphide
SBM: *Swachh Bharat Mission*
SDG: Sustainable Development Goal
WASH: Water, Sanitation and Hygiene
